# Supplementary material for: uORFlight: a vehicle toward uORF-mediated translational regulation mechanisms in eukaryotes
Source: Database (Oxford). 2020 Mar 13;2020:baaa007. doi: 10.1093/database/baaa007 (PMC7068905; doi:10.1093/database/baaa007)
Supplement: Supplementary_Information-pGX-5Dual-200108_baaa007 [file supplementary_information-pgx-5dual-200108_baaa007.docx]

**Supplementary Method: Ligation-independent cloning of Dual-LUC vector**

**Primer design for**  Dual-Luciferase vector pGX-5Dual (yellow means 5’ leader)

Target-F: 5’ CGA CGA CAA GAC CGT +++

Target-R: 5’ GAG GAG AAG AGC CGT +++

**LIC-PCR fragment**

Gel-purified PCR product 50 ng/kb (e.g. ~1 kb, add 50 ng)

10*Cutsmart buffer 1 μl

dATP (Promega, 100 mM) 0.5 μl

T4 DNA polymerase (NEB, M0203) 0.5 μl

H_2_O to 10 μl

12 ℃/30’, 75 ℃/20’

**LIC-Vector**

Plasmid (pGX-5Dual) 2 μg

10*Cutsmart buffer 10 μl

ApaI (NEB, R0114) 3 μl

H_2_O to 100 μl

25 ℃/60’, 65℃/20’ (~600 bp cut off, checking by running gel is optional)

**Then add:**

dTTP (Promega, 100 mM) 5.6 μl

T4 DNA polymerase (NEB, M0203) 3 μl

12 ℃/30’, 75 ℃/20’

**LIC ligation (**LIC-Vector : LIC-PCR fragment~1 : 3)

LIC-Vector 2.5 μl

LIC-PCR fragment 2.5 μl

75 ℃/5’, 22 ℃/10’

**Transformation and colony PCR**

1. Use 1-2 μl ligation product to transfer ccdB sensitive *E.coli* Strain, such as TOP10 and DH5a;
2. Select on Kan50 LB plate;
3. Colony PCR with Target-F/Target-R (5’leader plus 30 bp) or Target-F/OWH16 (5’TCAGCGTAAGTGATGTCCACCTCG3’; 5’leader plus 221 bp);
4. Sanger sequencing with OWH16 from LUC to 5’ leader：

TCAGCGTAAGTGATGTCCACCTCGatatgtgcatctgtaaaagcaattgttccaggaaccagggcgtatctcttcatagccttatgcagttgctctccagcggttccatcttccagcggatagaatggcgccgggcctttctttatgtttttggcgtcttccatggtagggtcttgtcgtcgaaggtacctGAGGAGAAGAGCCGT+++ACGGTCTTGTCGTCG

pGX-5Dual: A *ccdB* gene contained vector to be maintained in DB3.1 or ccdB survival strain; kan50 LB
